# Design and Development of an Internationally Applicable Educational Video to Increase Community Awareness in Regions with High Prevalence of Melioidosis and Diabetes

**DOI:** 10.4269/ajtmh.22-0024

**Published:** 2023-01-16

**Authors:** Shalisa C. Maisrikrod, Mathew Currie, Brenda L. Govan, Robert E. Norton, Bart J. Currie, Natkunam Ketheesan, Mark Mayo

**Affiliations:** ^1^ Royal Brisbane and Women’s Hospital, Herston, Australia;; ^2^ College of Medicine and Dentistry, James Cook University, Townsville, Australia;; ^3^ OneT Creations, Townsville, Australia;; ^4^ Chancellory, James Cook University, Townsville, Australia;; ^5^ College of Public Health, Medical and Veterinary Sciences, James Cook University, Townsville, Australia;; ^6^ Townsville University Hospital, Townsville, Australia;; ^7^ Faculty of Medicine, University of Queensland, Brisbane, Australia;; ^8^ Royal Darwin Hospital, Darwin, Australia;; ^9^ Menzies School of Health Research, Charles Darwin University, Darwin, Australia;; ^10^ School of Science & Technology, University of New England, Armidale, Australia

## Abstract

Melioidosis is a neglected tropical disease that causes high morbidity and mortality. Public health awareness is essential for both prevention and early detection of the infection. This project aimed to develop an internationally applicable educational tool to increase community awareness in regions with high prevalence of diabetes and melioidosis. The animation was created with international collaboration. Sixty-four delegates from different cultural backgrounds participated in the survey to evaluate the animation. Feedback was positive, with 85% agreeing that they would use this video for public education and 82% agreeing that the video was culturally appropriate to them in the context of their region. The animation was refined after feedback. To supplement the 3-minute animation, a 13-minute film footage of interviews with clinicians, researchers and patients was also created. These materials have been made available online through the International Melioidosis Network and can be readily downloaded or subtitled in any language using publicly available software, demonstrating the utility of developing low-cost adaptable health education material targeted for widespread use internationally

Melioidosis is caused by the Gram-negative bacterium *Burkholderia pseudomallei*, which is commonly found in soil in tropical regions around the world.^1^ It is an opportunistic pathogen typically infecting humans during periods of heavy rain and floods or through occupational exposure.^2^ It can be inhaled, ingested, or infect cutaneous lesions, manifesting as pneumonia, abscesses, or sepsis.^1^ Depending on the socioeconomic status of the region where the infection occurs, awareness of the disease, stage of presentation for medical treatment and the immune status of the host, mortality can range from as low as 10% to 15%^3^ to several folds more in countries of endemicity.^4^ Diabetes mellitus is a major risk factor affecting host susceptibility to intracellular infections including *B. pseudomallei*.^5^ Diabetes is present in 40% to 75% of patients with melioidosis.^3^^,^^4^ The rate of diabetes is increasing globally, and a double burden of disease exists in many melioidosis endemic countries.^6^ Melioidosis is an increasingly recognized disease worldwide. Currently, there is neither a vaccine nor cost-effective rapid diagnostics available. Therefore, prevention and early recognition through patient education are key aspects that avert serious clinical outcomes in melioidosis.^7^ More emphasis on developing appropriate educational material and programs to educate the population in endemic areas must be implemented.

The aim of this project was to design and develop an internationally applicable educational video to increase community awareness in regions with a high prevalence of both diabetes and melioidosis. Multimedia interventions have proven effective in improving management of chronic diseases and promoting awareness of infectious diseases.^8^^–^^11^ Thus far, information to the public on melioidosis is available in the form of factsheets and posters developed by various health providers or through local news outlets.^8^^,^^12^^–^^23^ Although these resources work well to target the local populations, the overall quality of such material can be variable. Developing high-quality material readily transferable internationally has its merits. As yet, no patient education material exists that is applicable across many cultures and easily adapted to multiple languages. Information presented in a multimedia format using text, still imagery, animations, film footage, and sound has the capacity to convey concepts effectively regardless literacy level, culture, or language.^24^^–^^26^ Videos are also advantageous for patient education because they can enhance explanations by health professionals.^8^

This original educational video on melioidosis consists of two components: an animation (5 minutes) and a compilation of filmed footage of interviews with patients, health professionals and researchers (13 minutes). Both components feature the following: an introduction to the infection, how infection with the bacterium occurs, signs and symptoms, risk factors with a particular emphasis on diabetes, information on diagnosis and management and a final highlight on the importance of prevention and early detection. These knowledge domains are consistent with existing resources available to the public.^12^^,^^13^^,^^17^^,^^18^ The animation provides colorful images and voiceover in simple language to aid in the explanation of complex concepts such as how the bacterium enters the body and how the immune dysfunction in diabetes could exacerbate the illness. The filmed interviews provide insights from individuals who themselves have experienced melioidosis, showing viewers real examples they can relate to, as well as health professionals who viewers may more readily trust.

The storyboard for the animation was developed following the assessment of imagery already available from different geographic regions and received input from all authors. A professional animator used Adobe Illustrator and Adobe After Effects (https://www.adobe.com/au/). Adobe Illustrator was used to create every asset, based on the initial storyboard (Figure 1). Adobe After Effects was then used to convert illustrations into motion graphics. For example, the globe is depicted as a long, flat layer; however the final video gives the illusion of a spinning world when it is in fact this single illustration moving from left to right. These details have created an animation with constant movement, always capturing the attention of the viewer.

**Figure 1.**
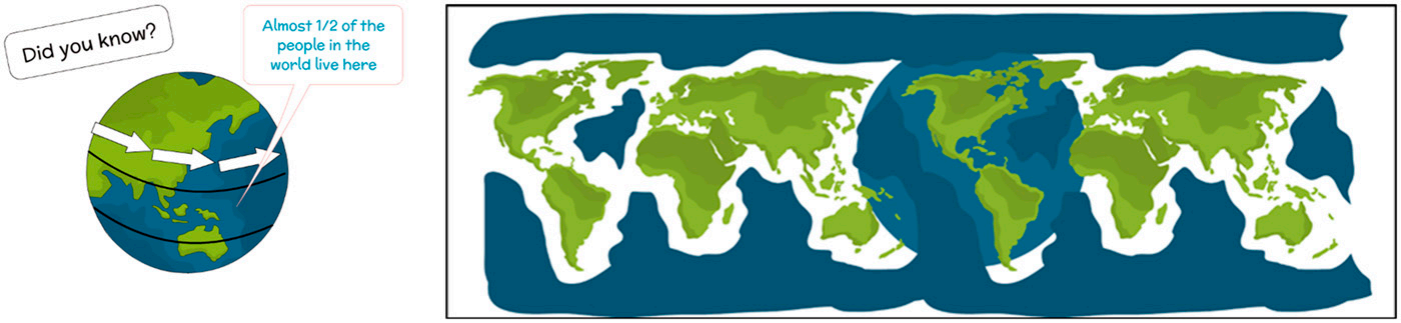
Initial storyboard design of animation with final graphic used for animation.

Interview footage was filmed across multiple endemic sites in northern Australia and Thailand. Patients who had previously had melioidosis were recruited for interviews through the network of melioidosis researchers. This enabled patients to share, in their own words, their experiences, explain what melioidosis is, and how to prevent infection. The primary author conducted all the interviews, asking the same set of open-ended questions in simple English, such as “What do you know about melioidosis?” Patients provided informed consent to enable sections of their interviews to be included in the final version. Refinement of the interview footage was conducted by the primary author with the aim of including varying explanations of the disease. For technical or general informational aspects of the video—that is, how melioidosis is diagnosed—researchers knowledgeable about melioidosis were interviewed. General questions regarding melioidosis were put to researchers, as well as questions specific to their field of work. Researchers from both Thailand and northern Australia were interviewed in their local language to provide some cultural and linguistic diversity. Filming was conducted in a cost-effective manner using a mobile phone, collapsible stand, and small Rode microphone that recorded the audio using a freeware application.

A survey was developed using Google Forms using Likert scales to assess the acceptability and applicability of the animation in an international context. The survey (Supplemental Material 1) was developed by the primary author and vetted by the coauthors. Professionals with a broad health and medical background and content experts on melioidosis attending the World Melioidosis Congress in Hanoi, Vietnam, in 2019 were opportunistically recruited to provide feedback and suggestions to improve the animation. The animation on two screens placed in high traffic areas accompanied by paper copies of the survey and a Quick Response code link to the survey that could be completed on their personal digital device. Responses from the paper survey were transcribed and compiled with the digital responses using Microsoft Excel. The Likert scales provided quantitative data, from which frequencies and percentages were calculated and graphically displayed (Figure 2). An open-ended question asking for suggestions to improve the video provided qualitative data. Answers were transcribed into NVivo and coded depending on the aspect of the video to which the feedback referred. Recurring feedback provided themes that influenced further edits to the animation.

**Figure 2.**
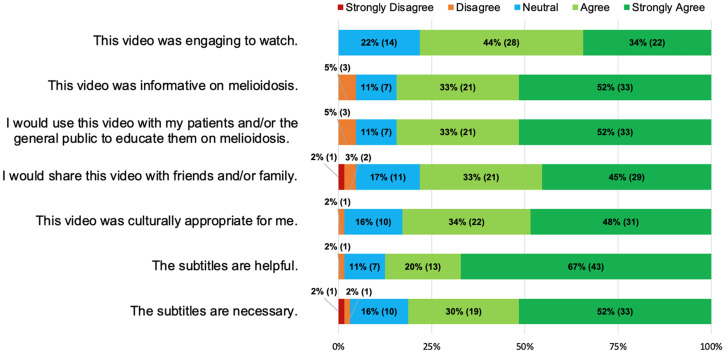
Feedback on the animation from international participants.

Sixty-four (20%) attendees participated in providing feedback with all having prior knowledge of melioidosis. Twenty different cultural and/or ethnic backgrounds were identified, with 58% (*N* = 37) being of an Asian cultural and/or ethnic background. Eighty percent (*n* = 51) of participants spoke more than one language. A third of participants (*n* = 21) preferred to educate the general population on melioidosis using only multimedia tools (Supplementary Figure 1A). Verbal explanation combined with multimedia education was preferred by 23% of participants, with verbal explanation preferred by 17%. Only 12% (*n* = 8) preferred all three methods (written, verbal, and multimedia) to educate the public on melioidosis. When asked if the participants would to use this animation produced for patient and/or public education on melioidosis, 85% (*n* = 54) responded in the affirmative (“agree” or “strongly agree”). A majority (82%; *n* = 53) of participants felt that the video was culturally appropriate, with 16% (*n* = 10) being neutral. More than two-thirds of the participants (*n* = 50) agreed that the video was engaging to watch. Seventy percent (*n* = 45) stated they liked the depth of information and detail as is, with 85% (54) participants agreeing that the animation was very informative. Most participants (87%, *n* = 56) agreed that the subtitles were necessary. Fifty-eight percent of participants (*n* = 37) would have preferred the animation to be shorter.

Overall, the animation was well-received, and feedback was largely positive. Most participants found the video to be engaging and informative. Participants agreed with this project’s approach, stating that they preferred to use multimedia tools to educate patients with melioidosis and communities in high-risk regions, with many stating that they would use this video for future patient education purposes. A recurring theme from the survey was to remove the lengthy explanation regarding the immune system explanation and the diabetes-specific information unless showing the video to people with diabetes. This led to a final version only 3.5 minutes in length. The filmed footage of interviews with patients, health professionals and researchers (13 minutes) has also been provided as a standalone component for those seeking further information. Although a great strength of this project was that the international participants who provided feedback were from melioidosis endemic regions, a limitation was that they were delegates at the international conference on melioidosis. All delegates at the conference were researchers, clinicians, or healthcare professionals with a comprehensive baseline knowledge of melioidosis. Therefore, it was assumed that change in the viewers’ level of knowledge regarding the disease would be insignificant before and after viewing the animation and therefore did not warrant assessment. Although multimedia educational materials have been shown to be advantageous compared with printed material in the initial phase of patient education, take-home materials are most important in reinforcing messages for long-term information retention.^27^^,^^28^ Take-home materials could be a link to the video or print information designed with the same graphics and messages of the video.

This project has demonstrated the plausibility of creating a low-cost but effective multimedia animation and video on a tropical disease that could be adapted for use in any region. The video is now available online (International Melioidosis Network)^29^ in different formats with subtitles in English and Thai that can be freely downloaded. The addition of subtitles or voice-over in different languages, can be done using specific software that can be freely obtained. Further evaluation of the education material in the field in different communities where melioidosis is endemic with varying levels of education is required to assess the educational value of this video. However, the material that has been produced in this project can serve as a template for even more effective educational tools.

## Supplemental files


Supplemental materials
