# Supplementary material for: Design and Development of an Internationally Applicable Educational Video to Increase Community Awareness in Regions with High Prevalence of Melioidosis and Diabetes
Source: Am J Trop Med Hyg. 2023 Jan 16;108(3):503–6. doi: 10.4269/ajtmh.22-0024 (PMC9978557; doi:10.4269/ajtmh.22-0024)
Supplement: Supplementary file 1 [file tpmd220024.SD1.pdf]

Supplement 1. Melioidosis Patient Education Videos Feedback Survey

1. Which patient education material on melioidosis did you view?
    a. Filmed interviews.
    b. Animation.
2. What is your cultural and/or ethnic background?
3. What is your primary language?
4. Can you speak more than one language?
    a. Yes.
    b. No.
5. If yes, please specify what other languages you can speak:
6. How do you prefer to educate people and/or patients on melioidosis?
    a. Verbal explanation.
    b. Providing written information.
    c. Showing multimedia information.
    d. Other.
7. Are there any particular words or phrases you use to explain melioidosis?
8. Please place a point on the scale from 1 (Strongly Disagree) to 5 (Strongly Agree) based on how much you agree with the following statements:
    a. This video was engaging to watch.
    b. This video was informative on melioidosis.
    c. I would use this video with my patients and/or the general public to educate them on melioidosis.
    d. I would share this video with friends and/or family.
    e. This video was culturally appropriate for me.
    f. The subtitles are helpful.
    g. The subtitles are necessary.
9. What would you suggest to improve the quality of this video?
10. I would prefer if this video was:
    a. Shorter.
    b. Longer.
    c. I liked the length as is.
11. I would prefer if this video had:
    a. Less information/detail.
    b. More information/detail.
    c. I liked the depth of information and detail as is.
12. On a scale from 1 (Definitely Note) to 5 (Definitely), how likely are you to use this video for patient and/or public education on melioidosis?

How do you prefer to educate people and/or patients on melioidosis?

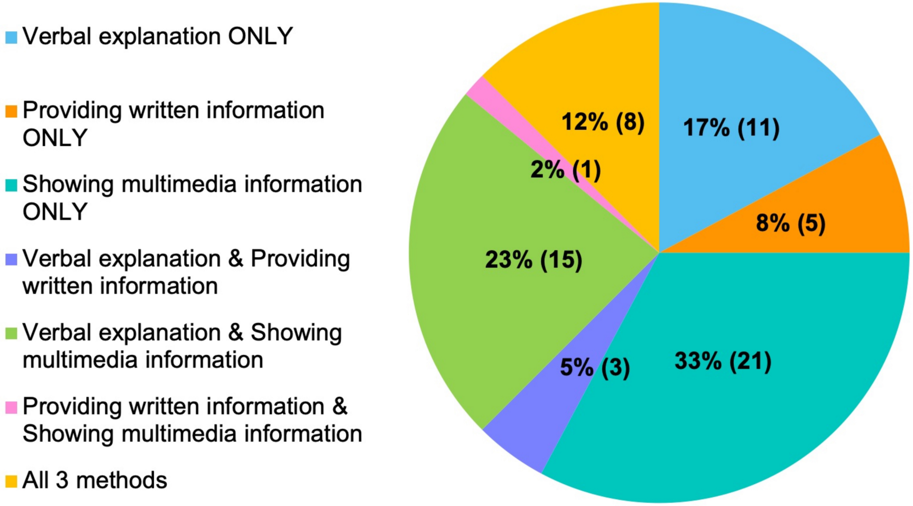

I would prefer if this video had...

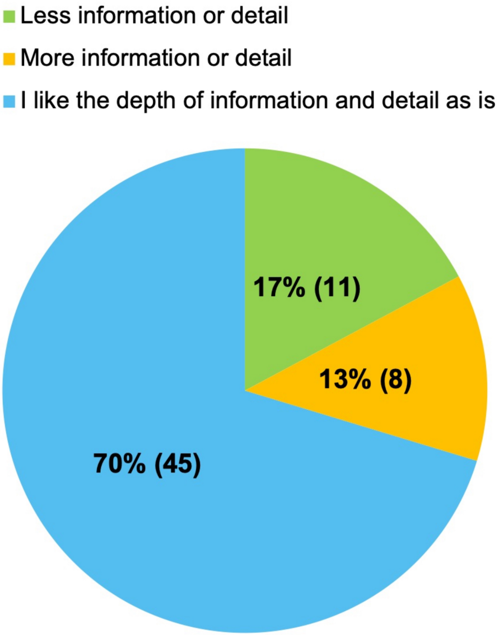

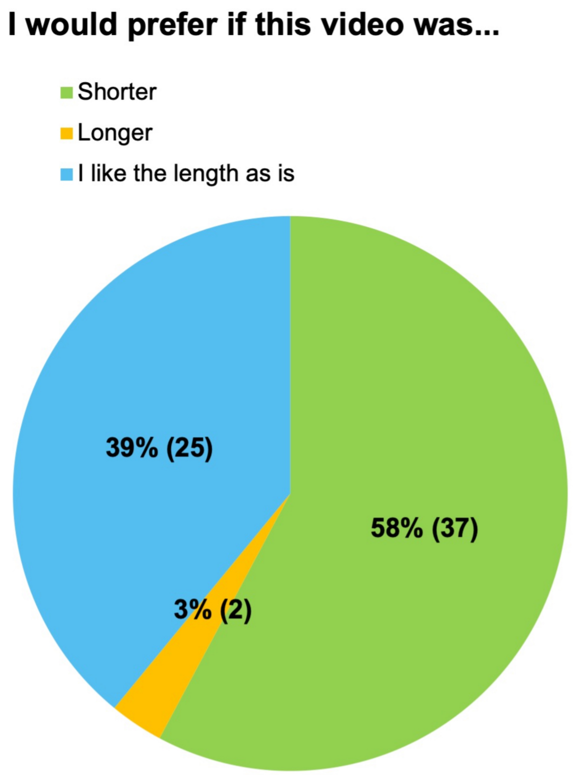
I would prefer if this video was...
Shorter
Longer
I like the length as is
39% (25)
58% (37)
3% (2)
